# Editorial: The future of andrology and infertility

**DOI:** 10.3389/fruro.2024.1480238

**Published:** 2024-09-27

**Authors:** Giorgio Ivan Russo, Ionnis Sokolakis, Giovanni Cacciamani, Andrea Cocci

**Affiliations:** ^1^ Department of Urology, University of Catania, Catania, Italy; ^2^ School of Medicine, Faculty of Health Sciences, Aristotle University of Thessaloniki, Thessaloniki, Greece; ^3^ Keck School of Medicine, University of Southern California, Los Angeles, CA, United States; ^4^ Urology section, University of Florence, Florence, Tuscany, Italy

**Keywords:** LUTS, HIV, male infertility, sperm, COVID, erectile and ejaculatory dysfunction

The rapid evolution of andrology and infertility over the past decade has sparked numerous debates and groundbreaking discoveries (1, 2). In this Research Topic, we bring together seven articles that collectively explore the forefront of male infertility, offering new insights and challenging existing paradigms.

The COVID-19 pandemic has had profound impacts on the management of chronic diseases, forcing healthcare systems to adapt to new challenges. This Research Topic presents seven studies that explore various facets of chronic disease management and male health, highlighting both the immediate effects of the pandemic and emerging trends in medical research.

The first study focuses on how the COVID-19 lockdown affected medication adherence among patients with lower urinary tract symptoms (LUTSs) due to benign prostatic hyperplasia (BPH). The study found that 13.25% of the patients experienced treatment interruptions during the lockdown, which led to a slight increase in symptoms and hospitalization rates. This highlights the critical need for telehealth strategies to ensure continuous care for chronic conditions, even during global crises (Morgia et al.).

Shifting focus to erectile dysfunction (ED), the second study explores the correlation between serum folic acid (FA) levels and penile arterial peak systolic velocity (PSV), a measure of endothelial function in the penile artery. The study suggests that lower FA levels are significantly correlated with reduced PSV in patients with arteriogenic ED, identifying FA deficiency as an independent risk factor for the condition (Feng et al.).

The third study delves into the relationship between physical activity and testosterone deficiency (TD). Using data from the National Health and Nutrition Examination Survey (NHANES), the study reveals a U-shaped relationship between physical activity levels and the risk of TD. While light to moderate physical activity is associated with a lower risk of TD, high-intensity activity surprisingly correlates with a higher risk, suggesting a need for balanced exercise routines in managing testosterone levels (Wu et al.).

In the fourth study, researchers examined the incidence of ED among sexual minority men (SMM) living with and without HIV over a 12-year period. The study found that SMM living with HIV had a significantly higher incidence of ED compared to those without HIV, particularly when other factors like age, diabetes, and cumulative use of cigarettes and antidepressants were considered. This underscores the importance of comprehensive management strategies for this population, including careful monitoring of comorbidities (Mustapha et al.).

The fifth study investigates the impact of paternal age on clinical pregnancy and perinatal outcomes among patients undergoing *in vitro* fertilization (IVF). The results indicate that paternal age over 45 years is associated with lower live birth and clinical pregnancy rates, emphasizing the need for age-related considerations in reproductive counseling and treatment planning (Gao et al.).

In the sixth study, a bibliometric analysis was conducted to assess the evolving research landscape of oxidative stress (OS) and male fertility. The study identified emerging research trends, including the role of antioxidants and oxidative stress in embryonic development. With China leading in publication volume and the United States in centrality, the study highlights the growing global interest in this field and points to future research directions focused on DNA methylation, polyunsaturated fatty acids, and antioxidant health management (Du et al.).

Finally, the seventh study examines the prevalence and risk factors of erectile dysfunction among men living with HIV (MLHIV) in northern Tanzania. The study finds a high prevalence of ED (74.6%), with significant associations to factors such as age, lack of physical activity, depression, and adherence to antiretroviral therapy (ART). Together, these studies provide a comprehensive overview of the challenges and advancements in chronic disease management and male health, particularly in the context of the ongoing pandemic. They underscore the importance of continued research and the adaptation of healthcare strategies to meet the evolving needs of patients worldwide (Mbwambo et al.).

We invite readers to actively engage with the groundbreaking findings presented in this Research Topic. The studies included offer critical insights into various aspects of chronic disease management and male health, highlighting both the challenges faced and the innovative solutions emerging in response to these challenges. We encourage healthcare professionals, researchers, and policymakers to apply these findings to their practices and decision-making processes. Integrating these insights into clinical routines, particularly those concerning medication adherence, physical activity, and the management of comorbid conditions, can lead to more effective and holistic patient care.

We also urge researchers to build upon these studies by exploring new avenues of investigation. The diverse topics covered—from the impact of the COVID-19 pandemic on chronic disease management to the role of oxidative stress in male fertility—offer numerous opportunities for further research. By delving deeper into these areas, researchers can contribute to a more comprehensive understanding of these complex issues and drive advancements in treatment and patient outcomes.

By engaging with these findings, applying them in real-world settings, and contributing to ongoing research and discussions, we can collectively advance the field and enhance outcomes for patients worldwide. The contributions of this Research Topic underscore the importance of continued exploration and collaboration in addressing the complex issues at the intersection of chronic disease management and male health.
